# F48. RANDOMISED CONTROLLED TRIAL OF SOCIAL COGNITION INTERACTION TRAINING

**DOI:** 10.1093/schbul/sby017.579

**Published:** 2018-04-01

**Authors:** Frances Dark, James Scott, Andrea Baker, Stephen Parker, Anne Gordon, Ellie Newman, Victoria Gore-Jones, Sukanta Saha, Carmen Lim, David Penn

**Affiliations:** 1 Metro South Health Services; 2 The University of Queensland, Queensland Centre for Mental Health Research, Metro North Mental Health Service; 3 Queensland Centre for Mental Health Research; 4 Bayside Community Care Unit Redland Bay; 5 Metro North Mental Health Service; 6 Queensland Centre for Mental Health Research, Queensland Brain Institute; 7 University of North Carolina, Australian Catholic University

## Abstract

**Background:**

Enthusiasm for the importance of social cognition in schizophrenia has grown as research has revealed that it is more strongly related to functional outcomes than neurocognition. A promising therapy developed by Roberts and Penn is Social Cognitive Intervention Training (SCIT). This therapy is comprised of three phases (i.e., Introduction & Emotions, Figuring out Situations, Checking it out) administered in a group format.

**Objective:**

To evaluate the efficacy of Social Cognition and Interaction Training (SCIT) in improving social cognitive and social functioning deficits of patients with schizophrenia spectrum disorder compared with standard of care, Befriending Therapy (BT).

**Methods:**

A 10-week, single-blind, randomized controlled trial (RCT) of SCIT and BT was carried out in 120 patients with schizophrenia spectrum disorder. Primary outcome measure is the total score on the Bell Lysaker Emotion Recognition Task (BLERT) at 12 weeks. Mixed Model for Repeated Measures was used to analyse change in BLERT score from baseline to 3-month follow-up between SCIT and BT groups. Secondary measures of the study are improvements on the Social Functioning Scale, [1] [1] [1] Hinting Task, Social Skills Performance Assessment, Internal, Personal and Situational Attributions Questionnaire, and Meta Cognition Questionnaire.

**Results:**

Among 120 patients, the mean age (SD) was 36.8 years (10.4) and 71.7% were males. Of these, 59 were randomized to the BT group and 61 to the SCIT group. The mean age of participants was 36.8 years. 85.8% were receiving government benefit and 50% lived in supported housing. 71.7% were males. Pre/Post data will be presented on the 91 participants who completed the study. Results examining the primary outcome measure found there was insufficient evidence to conclude that the SCIT group was significantly different compared to BT group in terms of emotion recognition (BLERT scores) (SCIT vs BT change: 0.437, 95% CI: -0.14 to 1.01; P = 0.136). There was an overall effect of time where both treatments showed a steady improvement over time from baseline to endpoint and the effect was maintained at the three-month follow-up. There was no significant time x treatment group interaction which indicated that there was no difference in patterns of change in the treatment group over time. Data on secondary outcomes is currently being analysed.

**Discussion:**

In this medium sized RCT of social cognition interaction therapy that used an active control, (BT) and standardised measure of emotional recognition, (BLERT) we found no significant difference between the interventions in our primary outcome measure of emotional recognition. Improvement in emotional perception has been found in the majority of studies of social cognitive interventions for schizophrenia. More specifically our results differ to those of Hasson-Ohayon who found significant improvement in emotion recognition in a RCT of SCIT with social mentoring compared with social mentoring alone in people diagnosed with schizophrenia, schizoaffective disorder, depression or bipolar disorder (Hasson-Ohayon 2014). This study is the largest RCT of SCIT to find a negative result in regards to emotion perception.

